# Changes Occurring on the Activity of Salivary Alpha-Amylase Proteoforms in Two Naturalistic Situations Using a Spectrophotometric Assay

**DOI:** 10.3390/biology10030227

**Published:** 2021-03-16

**Authors:** María D. Contreras-Aguilar, Sandra V. Mateo, Fernando Tecles, Christophe Hirtz, Damián Escribano, Jose J. Cerón

**Affiliations:** 1 Interdisciplinary Laboratory of Clinical Analysis (Interlab-UMU), Veterinary School, Regional Campus of International Excellence ‘Campus Mare Nostrum’, University of Murcia, 30100 Murcia, Spain; mariadolores.contreras@hotmail.com (M.D.C.-A.); sandra.valverde@um.es (S.V.M.); ftecles@um.es (F.T.); det20165@um.es (D.E.); 2 IRMB-PPC, CHU Montpellier, Montpellier University, 34295 Montpellier, France; christophe.hirtz@umontpellier.fr; 3 Department of Animal Production, Veterinary School, Regional Campus of International Excellence ‘Campus Mare Nostrum’, University of Murcia, 30100 Murcia, Spain

**Keywords:** salivary alpha-amylase, proteoforms, glycoprotein depletion, physical effort, psychological challenge

## Abstract

**Simple Summary:**

Salivary alpha-amylase (sAA) is considered a biomarker of acute stress since this enzyme is released in saliva after autonomic nervous system activation, in response to psychological or physical stress situations. This enzyme has different isoforms that could be differentially expressed depending on the stressful situation. The aims of the present research were (1) to develop and validate an easy and fast method to estimate the activity of the major sAA proteoforms (both non-glycosylated and glycosylated proteoforms) in saliva samples, and (2) to evaluate the possible changes occurring in the activity of both proteoforms when measured by this method in two different stress models (physical effort and psychological challenge). This new method was precise and, when applied to the different stress models, allowed to detect changes of different magnitudes in both proteoforms. Therefore, this research opens a new field for the evaluation of isoforms of sAA as potential biomarkers of stress.

**Abstract:**

This study aimed to evaluate the changes in the activity of total salivary alpha-amylase (TsAA) and both the non-glycosylated and glycosylated salivary alpha-amylase proteoforms (NGsAA and GsAA, respectively) in physical and psychological stress models, estimated using a simple and easily set-up method. The method used was a spectrophotometric assay with 2-chloro-4-nitrophenyl-α-D-maltotriose (CNPG3) as a substrate, incubated with Concanavalin A (ConA) to remove most of the glycosylated protein from the sample. This method allowed the measurement of TsAA and estimation of NGsAA and GsAA activities with imprecision lower than 10%. When this method was applied to two different stress models, differences in the responses of the proteoforms were observed, with the NGsAA activity showing changes of higher magnitude after stress induction than the GsAA activity, and the highest correlation with the State–Trait Anxiety Inventory Scale in the Trier Social Stress Test (TSST). In conclusion, the activity of the two main sAA proteoforms can be easily estimated in saliva, and their measurement can provide additional information on TsAA activity in physical or psychological stress situations.

## 1. Introduction

Salivary alpha-amylase (sAA, EC 3.2.1.1) is described as the major protein in human saliva, having importance in catalyzing the hydrolysis of 1,4-α-glucosidic linkages in starch and other polysaccharides to glucose and maltose [1,2]. It is also a predictive marker of stress [2,3], since it is secreted directly by salivary glands in response to autonomic nervous system (ANS) activation, increasing concentration in response to situations of psychological or physical stress [4,5].

The AMY1 genetic locus, which is placed in the chromosomal locus 1.21.1 and contains the AMY1A, AMY1B, and AMY1C genes, is involved in the synthesis of the sAA [6], producing 12 distinct phenotypes of the salivary sAA isoenzymes, all of which are included in the non-glycosylated (NGsAA) proteoform of 56 kDa [7]. However, additional sources of diversity by post-translational modifications like glycosylation, can produce other proteoforms resulting, for example, in the glycosylated (GsAA) proteoform at 59 kDa [7].

Recently, a study demonstrated that proteoform expression could change differently depending on the type of stressful situation and that the native non-glycosylated proteoform correlates with the total activity [8]. This study raised the interest in assessing total sAA (TsAA) and the GsAA and NGsAA proteoforms as stress biomarkers. However, these assessments are limited in routine practice due to their need for the use of technically complex gel separation methods that require at least one day for their completion. The possible application of methods that would not need gel separation and that could be easy and fast to perform would represent an advantage. Concanavalin A (ConA) binds glycosylated proteins [9], and the incubation of a saliva sample with ConA following centrifugation would remove most of the glycosylated protein from the supernatant. Therefore, the measurement of sAA in this supernatant would estimate the NGsAA, and the difference between the measurement of the TsAA of the sample without any treatment minus the estimation of NGsAA could be used to estimate the GsAA.

We hypothesized that GsAA and NGsAA proteoform activities could be estimated by a simple incubation assay using Concanavalin A (ConA), and that the joint measurements of TsAA and these proteoforms could contribute to better study and understanding of the response of sAA to different stress situations. The aims of this study were therefore (1) to develop and validate an easy and fast method of estimating the activity of GsAA and NGsAA in saliva samples; (2) to evaluate the possible changes occurring in the activity of the GsAA and NGsAA proteoforms when measured by this method in two different stress models (physical effort and psychological challenge).

## 2. Materials and Methods

The stress models used were physical effort and psychological challenge. Participants in both stress models did not ingest any stimulating substances or alcohol 24 h before the session, were not allowed to eat or consume dairy products 1 h before the beginning of saliva collection, and reported not having oral or systemic diseases. Also, they were all informed about the procedure, sampling methods, and objectives of the experiments. Additional informed consent was obtained from all individual participants for whom identifying information is included in this article. This project was approved by the Murcia University Ethics Committee (number reference: 1349/2016).

The physical effort consisted of a CrossFit workout of the day (WOD) performed in a CrossFit training center. Participants (*n* = 10) were all males (Table 1), with the inclusion criteria described by Timón et al. [10], concerning no disease or injuries, and experience in CrossFit training. The maximum load lifted (one repetition maximum, 1RM) in the power clean test was recorded, which was previously obtained from the participants following the protocol developed by Faigenbaum et al. [11]. The exercise consisted firstly of a warm-up of 5 × 3 sets of overhead squats (OHS) for 5 min, and three different WODs in rounds for time (RFT). WOD1 consisted of two rounds of OHS (min 40 kg), and toes-to-bar (T2B) with increasing repetitions (2-2, 4-4, 6-6, 8-8, 10-10). WOD2 consisted of two rounds of power clean (min 40 kg) and pistols with increasing repetitions (2-2, 4-4, 6-6, 8-8, 10-10). WOD3 consisted of two rounds of OHS (min 40 kg), T2B, power clean (min 40 kg), and pistols with increasing repetitions (2-2, 4-4, 6-6, 8-8, 10-10). All repetitions had to be completed before beginning the next exercise. Each round lasted 2 min, with rests of 2 min between rounds. The total time to perform the exercise was 2328 ± 107.33 s. Saliva samples were obtained 5 min before the exercise (Tb), between rounds during WOD2 (T + 01), after completion of the exercise (T + 02), and 10 min after (T + 10). The experimental procedure was performed between 18:00 and 20:00 in two groups of classes (5 + 5 participants in each class). To evaluate the rating of perceived exertion (RPE), participants filled out the OMNI-Resistance Exercise Scale (OMNI-RES) at T + 01 and T + 02 validated for resistance exercise [12], consisting of 10 increasing options from 1 (extremely easy) and 10 (extremely hard) as described previously [12].

The psychological challenge was performed by 18 female university students (average age = 26.2 ± 6.26 years, body mass index [BMI] = 21.8 ± 2.65 kg/m^2^), who were submitted to a laboratory social stressor (Trier Social Stress Test, TSST) [3]. The procedure was performed as previously described [8,13], and saliva samples were obtained while each participant rested in the isolation room for 5 min before the interview (Tb), just after the arithmetic task (T + 0), and 15 min later (T + 15). The procedure lasted 30 min, and the participants performed the experimental procedure between 17:30 and 18:30. The Spanish version of the State–Trait Anxiety Inventory (STAI-P) [14,15] was filled out by the students at Tb and T + 0 to evaluate the anxiety suffered by the subjects. The STAI-P is composed of two scales evaluated independently: the trait form (STAI-P Trait), and the state form (STAI-P State). Each form evaluates anxiety by 20 items using a four-point graded response scale ranging from “almost never” or “not at all” (0), to “almost always” or “very much” (3), respectively.

In all stress models, saliva samples were collected by passive flow over 1 min, using 5 mL standard micro-centrifuge polystyrene tubes with round bottoms (12 × 75 mm) as described by Contreras-Aguilar et al. [8]. Samples were stored on ice until arrival at the laboratory in less than 3 h [16]. Once there, the saliva samples were centrifuged (4500× *g* for 10 min at 4 °C) [17], and the supernatant was obtained. Samples with evident blood contamination assessed by visual inspection were excluded from the study. Saliva samples were stored at −80 °C until analysis (less than one month) [18].

The amylase measurements were performed in various steps:

1. Measurement of sAA in the sample using a colorimetric commercial kit (Alpha-Amylase, Beckman Coulter Inc., Fullerton, CA, USA) following the International Medicine (IFCC) method [19] that was previously validated for TsAA [20]. It uses 2-chloro-4-nitrophenyl-α-D-maltotrioside (CNPG3) as an enzyme substrate that directly reacts with sAA, producing 2-chloro-4-nitrophenol (CNP). The resulting absorbance increase per minute is directly related to sAA activity in the sample. This gave the value of TsAA.
2. Depletion of the sample by the lectin ConA, which provided a fast glycoprotein depletion of the sample. For this step, a fixed volume of sample was mixed in a 1:16 ratio with ConA (Cocanavalin A-Sepharose^®^ 4B, Sigma-Aldrich, St. Louis, MO, USA) and incubated at 4 °C for 15 min. Then, the supernatant was removed and analyzed for sAA activity corresponding to the non-glycosylated proteoform.
3. The difference between the sAA measured in step 1 (TsAA activity) and the sAA measured in step 2 (NGsAA activity) was considered as GsAA activity. In all cases, sAA activity was expressed as IU/mL [21].

To validate the glycoprotein depletion procedure, we first evaluated the changes of the proteoforms in a western blot (WB) after glycoprotein depletion. For this purpose, two WBs were performed on two pools of saliva samples before (B) and after (A) applying the sample depletion procedure. The pools of saliva were made by mixing an equal quantity of saliva (100 μL) from (1) three individuals who participated in the psychological stress experiment described above at T + 0 (pool 1), and (2) from three individuals who participated in the physical stress model described above at T + 01 (pool 2). The specimens included in each saliva pool were selected based on having the highest TsAA activities. Polyacrylamide gels containing 0.1% (*w/v*) sodium dodecyl sulfate (SDS-PAGEs) were made according to the methodology described by Laemmli [22], with a separating one-dimension gel prepared in 12% (*w/v*) and a stacker gel prepared in 4% (*w/v*). Samples were added to the gels without and with the glycoprotein depletion procedure described above, at 3 μg of total protein per lane. Then, the proteins from the SDS-PAGEs were transferred to a nitrocellulose membrane (Bio-Rad Laboratories Inc., Hercules, CA, USA). A rabbit polyclonal antibody against human sAA produced by the researchers’ laboratory [8] at 1:6000 dilution was used as a primary antibody, while horseradish peroxidase (HRP)-conjugated goat polyclonal anti-rabbit antibody (ab 6721, Abcam, Cambridge, UK) at 1:2000 was employed as a secondary antibody, which was detected using a Pierce ECL2 kit (Pierce, Thermo Fisher Scientific, Rockford, IL, USA). The WB images were obtained using an ImageQuant™ scanner (GE Healthcare, Uppsala, Sweden). Quantification of each protein band (μg) at 59 kDa and 56 kDa was estimated by comparison with a known quantity of a natural human sAA protein (77875, Abcam, Cambridge, UK), analyzed using ImageQuant™ TL 8.1 (GE Healthcare, Uppsala, Sweden). Total proteins were determined using Lowry’s method for all the pool saliva samples.

Later, analytical validation of the glycoprotein depletion procedure was made by calculating the imprecision of the procedure and estimating the accuracy of the NGsAA measurements by evaluation of linearity under dilution.

- Imprecision. Three saliva samples with different TsAA activity (A = 400.62 IU/mL; B = 183.58 IU/mL; C = 171.18 IU/mL) underwent the procedure three times. The within-run coefficient of variation (CV) was then calculated as the percentage of the standard deviation (SD) of the replicates, divided by the mean.
- Accuracy. Two samples with different NGsAA activities were serially diluted (1/2, 1/4, 1/8, 1/10, 1/20) with deionized water. Then, linear regression between the observed and the expected results was performed, and the slope, y-intercept, and R2 were calculated.

Finally, a test was performed to evaluate if the lectin ConA binds specifically to GsAA. For this purpose, three saliva samples with different TsAA activity (specimen 1 = 368.5 IU/mL; specimen 2 = 133.0 IU/mL; and specimen 3 = 39.9 IU/mL) were treated using the ConA depletion procedure. The supernatant was separated and analyzed for sAA activity corresponding to NGsAA proteoform activity. After removing this supernatant and isolating the sediment Con-A Sepharose, a solution of 1M methyl mannopyranoside (MMp, Methyl α-D-mannopyranoside, Sigma-Aldrich, St. Louis, USA) [23] at a dilution ×100 times the sample volume was added and incubated at 4 °C for 10 min. This supernatant was then analyzed for sAA activity corresponding to the GsAA proteoform activity. The difference between the TsAA and the sum of both supernatants was calculated. The 1:100 sample dilution with MMp was selected according to a previous experiment (Table S1) where the sAA activity after adding different sample dilutions (1:12.5, 1:25, 1:50, 1:100, and 1:200) of MMp was compared using Fisher’s test. The dilution selected for the complete sAA separation from ConA was 1:100, because it showed a significant increase in separated sAA activity compared to the 1:12.5 dilution and yielded similar activities as the 1:200. Therefore, it would indicate that this sample dilution with MMp added to the ConA produced the maximum separation without increasing sample dilution.

The arithmetic means, SD, and CVs in the validation of this method were calculated using routine descriptive statistical procedures by spreadsheet (Excel 2019, Microsoft Corporation, Redmond, Washington, DC, USA). The percentage of difference in the activity or amount of each WB band before and after sample treatment was calculated using the formula: [((B − A) × 100)/B], where B and A are the values obtained before and after the treatment, respectively. The normality of distribution for sAA activities in the stimulation models and the results obtained in the STAI-P questionnaires were assessed using the Shapiro–Wilk test. Data showing non-normal distribution (NGsAA and GsAA in the physical effort, and TsAA, NGsAA, and GsAA in the psychological challenge) were base-e log-transformed by applying the formula ln (x + 1) before the statistical analyses [4,24], which restored normality. Then, a one-way ANOVA of repeated measures was calculated, and Tukey’s multiple comparison test was used to assess the significant changes at each time, for the sAA activities. A paired Student’s t-test (2-tailed) was used to determine if the values obtained from the STAI-P State questionnaires at Tb and T + 0 in the psychological challenge were statistically different. Results were described as mean or median based on their normality distribution. In order to guarantee a correct significance level (α = 5%, *p* < 0.05) and the power required (1 − β ≥ 80%) with the number of participants evaluated for each stress model, a post hoc analysis using a standalone power program for statistical testing (G-Power) [25] was employed using the means and SD of the sAA activities (TsAA, NGsAA, and GsAA). The Spearman r was calculated between the sAA activities in the psychological challenge at Tb and T + 0, and the STAI-P questionnaires in order to determine whether there was a correlation between them. An r value from 0.90 to 1 was considered to have a very high correlation, 0.70 to 0.90 had a high correlation, 0.50 to 0.70 had a moderate correlation, 0.30 to 0.50 had a low correlation and less than 0.30 had little if any correlation [26]. The significance level used in each case was *p* < 0.05. These statistical analyses were calculated using Graph Pad Prism 6 (GraphPad Software, San Diego, CA, USA).

## 3. Results

### 3.1. Validation of the Glycoprotein Depletion Procedure

**Figure 1:**
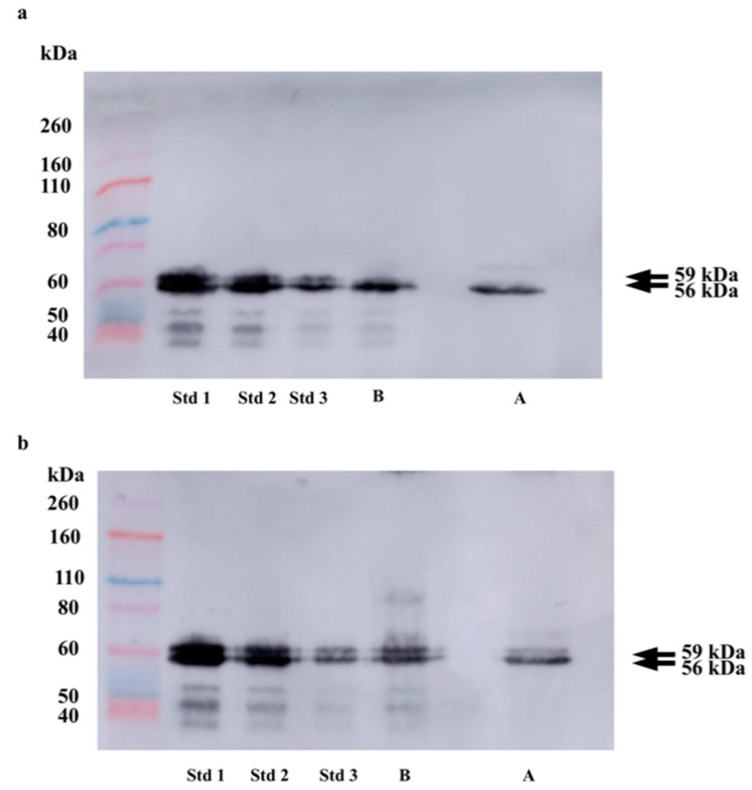
Western blots for salivary alpha-amylase (sAA) detection in two pool saliva samples from three participants during psychological (**a**) and physical (**b**) stress models, before (B) and after (A) a glycoprotein depletion procedure. Samples from the psychological stress model were obtained during the Trier Social Stress Test (TSST) just after the arithmetic task (T + 0). Samples from the physical stress model were obtained between rounds of exercise during a CrossFit workout of the day (WOD) (T + 01). Saliva samples were added at 3 μg per lane. The standard (Std) was natural commercial purified human salivary alpha-amylase (sAA) protein (77875, Abcam, Cambridge, UK) of known quantity (Std 1 = 3.5 μg; Std 2 = 1.75 μg; Std 3 = 0.44 μg). Molecular weight markers were Novex Sharp Pre-Stained (Invitrogen, Carlsbad, CA, USA). The arrows on the side show the bands at 59 kDa (up) and 56 kDa (down) where the native sAA proteoforms, glycosylated sAA (GsAA), and non-glycosylated sAA (NGsAA), are localized, respectively.

Figure 1, Figure S1, and Table 2 show the changes in the sAA proteoforms after the glycoprotein depletion in a WB. Reductions of 80.4% and 70.2% were observed in the band at 59 kDa after applying the glycoprotein depletion procedure in the two saliva samples analyzed.

**Figure 2:**
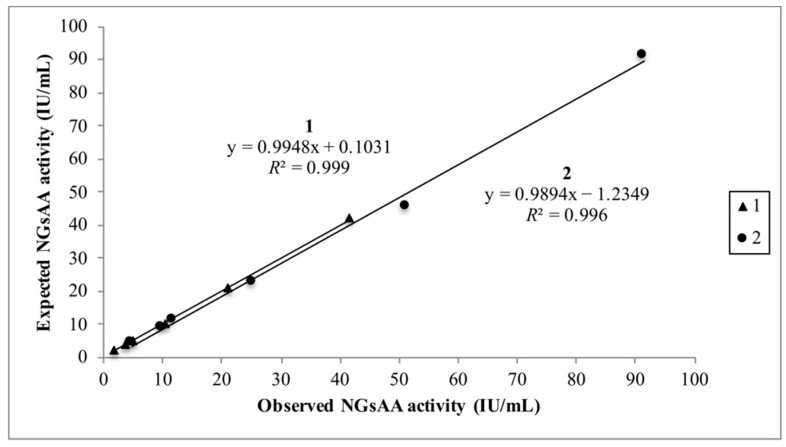
Linearity under dilution in the NGsAA activity measurements after the glycoprotein depletion procedure. A linearity under dilution study was performed in two saliva samples with different NGsAA activities (specimen 1 = 41.87 IU/mL; specimen 2 = 91.44 IU/mL). The x-axis expresses activity or concentration measured and the y-axis expected level at the particular dilution. *R*^2^ = coefficient of determination of linear correlation.

The depletion procedure (Table 3) to estimate NGsAA activity in three saliva samples showed an imprecision lower than 10%. Linear regression analysis (Figure 2) showed *R*^2^ higher than 0.995 when two saliva samples were measured for amylase enzymatic activity after the glycoprotein depletion procedure (1 = 41.87 IU/mL; 2 = 91.44 IU/mL), with slopes [0.99 (0.98; 1.01) and 0.99 (0.90; 1.08), respectively] not significantly different from one, and intercepts [0.10 (−0.16; 0.37) and −1.24 (−5.24; 2.76), respectively] not significantly different from zero.

The sums of the sAA activities in (1) the supernatant after the application of the depletion treatment using ConA to obtain the NGsAA and (2) after the application of MMp to the ConA to obtain the GsAA were similar to the initial TsAA values (Table 4).

### 3.2. Activity Results in the Stress Models

#### 3.2.1. Physical Effort

Two participants were excluded from the study because one had to leave after finishing the exercise, and in another case, the volume of saliva obtained at T + 01 was not enough. Therefore, the physical effort test was ultimately performed using eight participants.

**Figure 3:**
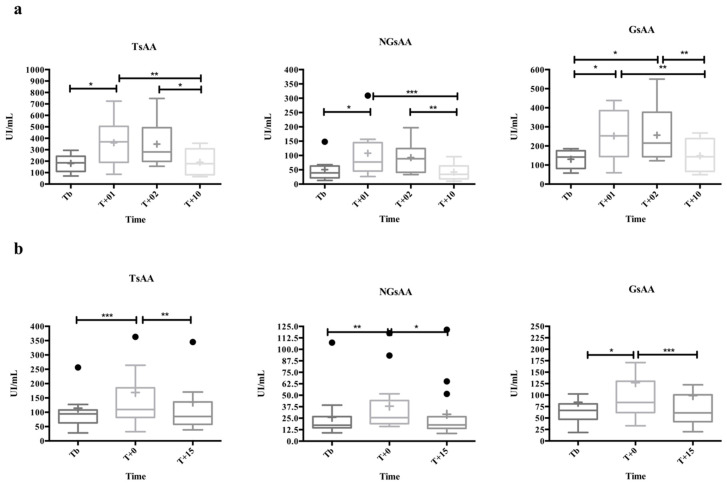
Total non-glycosylated and glycosylated sAA (TsAA, NGsAA, and GsAA, respectively) activity results in two different stimulation models. (**a**) Physical effort (*n* = 8), based on a CrossFit WOD, five min before the exercise (Tb), between rounds of WOD2 (T + 01), after completion of the exercise (T + 02), and 10 min after (T + 10). (**b**) Psychological challenge (*n* = 18), based on the standardized Trier Social Stress Test (TSST), 5 min before the test (Tb), just after (T + 0), and 15 min later (T + 15). The plot shows median (line within box), 25th and 75th percentiles (box), 5th and 95th percentiles (whiskers), and outliers (•). The cross inside the box shows the mean. Asterisks indicate significant post hoc difference (Dunn’s multiple comparisons test) with respect to the previous time: * *p* < 0.05; ** *p* < 0.01; *** *p* < 0.001.

Figure 3a shows the activity results from the CrossFit WOD model. The TsAA (F1.41,9.89 = 12.29, *p* = 0.003), NGsAA (F1.76,12.37 = 14.75, *p* < 0.001), and GsAA (F2.11,14.80 = 10.67, *p* = 0.001) activity significantly changed over time, increasing at T + 01 compared to Tb (2.0 mean-fold, *p* = 0.047; 1.9 median-fold, *p* = 0.030; and 1.8 median-fold, *p* = 0.049; respectively), and decreasing at T + 10 (−2.1 mean-fold, *p* = 0.009; −2.3 median-fold, *p* < 0.001; and −1.8 median-fold, *p* = 0.005; respectively) compared to T + 01. GsAA increased significantly at T + 02 compared to Tb (1.5 median-fold, *p* = 0.014), then decreased at T + 10 (1.5 median-folds, *p* = 0.004). The post hoc analysis computed a power of 83%, 93%, and 87% for the TsAA, NGsAA, and GsAA results, respectively.

The RPE measured from the participants between rounds of WOD2 (T + 01) and after completing the exercise (T + 02) reached values of 7.6 ± 0.48 and 6.8 ± 0.87, respectively.

#### 3.2.2. Psychological Challenge

Figure 3b shows the results obtained in the TSST. Significant changes were observed for TsAA (F1.99, 31.89 = 16.08, *p* < 0.001), NGsAA (F1.58, 22.14 = 7.27, *p* = 0.006), and GsAA (F1.46, 20.37 = 7.83, *p* = 0.006) activities. Total sAA (1.2 median-fold, *p* < 0.001), NGsAA (1.5 median-fold, *p* = 0.004), and GsAA (1.3 median-fold, *p* = 0.014) activities increased at T + 0 compared to Tb, then decreased at T + 15 compared to T + 0 for TsAA (−1.3 median-fold, *p* = 0.003), NGsAA (−1.4 median-fold, *p* = 0.026), and GsAA (−1.3 median-fold, *p* < 0.001). The post hoc analysis computed a power for the TsAA, NGsAA, and GsAA activities of 40%, 83%, and 30%, respectively.

The rate of state anxiety was significantly higher (*p* = 0.009) at T + 0 (24.8 ± 12.13) compared to Tb (16.4 ± 8.76). The mean STAI-P Trait obtained was 19.4 ± 10.85. Significant correlations were observed between the STAI-P State and TsAA and NGsAA (r = 0.40, 95% confidence interval [CI] 0.03–0.67, *p* = 0.030; r = 0.58, 95% CI 0.27–0.78, *p* < 0.001; respectively).

## 4. Discussion

In this study, a fast and easy procedure was used to estimate GsAA and NGsAA proteoform activities. Although in our case, part of the procedure was made in an automated biochemistry analyzer, this assay could be adapted to other equipment or formats such as plate readers or manual spectrophotometric analyzers.

The WB images demonstrate that the glycoprotein depletion treatment used in this study reduced more than 70% of the band at 59 kDa. The lack of total elimination of the 59 kDa band of the WB could have occurred because some parts of the glycosylated proteoform did not bind to the ConA [9]. The error of estimation carried out using the WB image could also have contributed [27]. The change in the 56 KDa band was less than 10%. This variation could be influenced by the error of estimation carried out using the WB image, and overall this result would indicate that the lectins did not produce a significant effect on the non-glycosylated proteoform.

The use of MMp can separate the GsAA bound to the ConA [28], and its activity together with the supernatant activity obtained after the depletion procedure was similar to the total activity of the samples. This finding could be additional evidence that the treatment used in our report can produce a separation between the GsAA and NGsAA proteoforms of sAA. Therefore, the arithmetic difference between the sAA activity before the treatment (TsAA activity) and after the depletion procedure (NGsAA activity) could be used to estimate GsAA.

The approximate percentages of GsAA and NGsAA activities related to TsAA after applying our depletion procedure were 75% and 25%, respectively. This would indicate that the GsAA proteoform plays a major role in the TsAA activity. This was an unexpected finding since NGsAA is the predominant proteoform of TsAA in terms of quantity, as can be observed in the results of our WBs, and as has been reported in other studies [7,29]. This is in line with the fact that in the case of sAA, the quantity of the enzyme does not correspond with its activity, as was previously indicated in another report [21]. Additionally, GsAA activity and concentration have been specifically described as showing a low correlation [30]. Further studies should be undertaken to clarify the divergence, but other factors could also be related, such as the different affinities of the substrates used for sAA activity measurement of the different proteoforms [9].

Both of the stress models performed in our study produced physical or psychological stress in the participants since the RPE results obtained in the CrossFit WODs were representative of an exercise intensity between “somewhat hard” and “hard” [12], and the social perceived state anxiety in the TSST showed significantly higher values after the time of stress than before. TsAA increased after the stressful situation in both models. However, when the sample treatment was applied, the estimation of NGsAA and GsAA activities provided interesting information in addition to TsAA in both cases. Overall, the NGsAA showed a higher magnitude of change in activity after stress induction than the GsAA activity did. NGsAA increases after different situations of sAA activation were previously reported [8]. Additionally, the NGsAA activity showed a higher correlation with the anxiety state than the TsAA activity did. The changes of a lower magnitude that GsAA showed at the stressful times in our tests could be explained because, after stress, there is an activation in the sAA salivary secretion by the salivary glands’ cells. This can lead to a decrease of its glycosylation in the endoplasmic reticulum and Golgi apparatus that is made as a previous step for this enzyme storage in the acini cells’ vesicles [31]. In the future, it would be of interest to evaluate the changes in both proteoforms in chronic stress situations.

Our study has some limitations. Although a correct power was achieved in the physical effort results and the results of NGsAA activity assay in the psychological stress test, it was not enough for the TsAA and GsAA activity. In addition, although this study allows us to indicate that our procedure of the estimation of sAA proteoforms can be applied to both sexes, the fact that we used different sexes in each model could limit the comparison of the results between them. Ideally, future studies should evaluate the differences between sexes in the response of the different sAA proteoforms on the same stress models and also on how different stress models can affect the same individuals. Furthermore, sAA has demonstrated a diurnal pattern [16]. Both experimental stress models were carried out during afternoons and at different times in order to adapt to participant availability. Therefore, differences due to the sAA diurnal pattern could not be avoided. The existence of circadian variations in this enzyme indicates that different behaviors of the sAA proteoforms observed in this study should not be extrapolated to another period of the day. Also, it is important to point out that the method used in our study provides an estimation and not the real values of the different sAA proteoforms.

## 5. Conclusions

In conclusion, this study developed and validated an innovative method that allows for the estimation of the activity of the two main sAA proteoforms (GsAA and NGsAA) quickly and easily. When applied to different stress models, this procedure allowed to gain information about changes in these sAA proteoforms. Therefore, it could be used, together with TsAA measurements, to obtain a more complete picture of the changes occurring in sAA under different stress conditions.

## Figures and Tables

**Table 1:** Description of participant characteristics (*n* = 10, males).

Variable	Mean ± SD ^1^
Age (years)	33.1 ± 5.19
Bodyweight (kg)	82.1 ± 7.59
Height (m)	1.8 ± 0.07
Body Mass Index	25.0 ± 2.29
1RM Power Clean (kg)	85.0 ± 11.02

^1^ Standard deviation (SD); one repetition maximum (1RM).

**Table 2:** Estimated concentration (µg) of bands of salivary alpha-amylase at 59 kDa and 56 kDa in the western blot (WB) and percentage (%) of difference before and after a glycoprotein depletion procedure. Saliva specimens were obtained from two pool saliva samples from three individuals who participated in a psychological stress test (the Trier Social Stress Test, TSST, specimen 1) just after the arithmetic task (T + 0), and in an exercise of CrossFit WODs (specimen 2) between rounds (T + 01), respectively. Pool samples were added at 3.0 μg per lane.

	TSST		CrossFit WODs	
	T + 0		T + 01	
	59 kDa	56 kDa	59 kDa	56 kDa
Before sample depletion	0.51	0.61	0.57	0.62
After sample depletion	0.10	0.67	0.17	0.56
% of difference	80.4	9.8	70.2	9.7

**Table 3:** Mean, standard deviation (SD) and coefficient of variation (CV) obtained in the imprecision study of the NGsAA measurement.

Saliva Specimens	Mean (IU/mL)	SD (IU/mL)	CV (%)
A	95.17	5.05	5.30
B	38.29	0.32	0.86
C	34.35	2.53	7.36

**Table 4:** Activity results of salivary alpha-amylase (sAA) in three saliva specimens with different activities measured before the depletion treatment (Total sAA, TsAA), in the first supernatant obtained after the depletion treatment corresponding to the non-glycosylated sAA (NGsAA) proteoform, and in the second supernatant obtained after adding 1M methyl mannopyranoside (MMp) at 1:100 sample dilution corresponding to the glycosylated sAA (GsAA) proteoform.

	Specimen 1		Specimen 2		Specimen 3	
	Activity Measurement	NGsAA + GsAA	Activity Measurement	NGsAA + GsAA	Activity Measurement	NGsAA + GsAA
TsAA (IU/mL)	368.5		133.0		39.9	
NGsAA (IU/mL)	69.8		21.7		6.9	
GsAA (IU/mL)	285.8	355.8	91.4	113.2	26.8	33.7

## Data Availability

No new data were created or analyzed in this study. Data sharing is not applicable to this article.

## Supplementary Materials

The following are available online at https://www.mdpi.com/2079-7737/10/3/227/s1, Table S1: Activity results of salivary alpha-amylase (sAA) in three saliva specimens with different activities measured before the depletion treatment (TsAA), in the first supernatant obtained after the depletion treatment corresponding to the non-glycosylated sAA (NGsAA) proteoform, and in the second supernatant obtained after adding a different sample dilution of 1M methyl mannopyranoside (MMp) corresponding to the glycosylated sAA (GsAA) proteoform. *p*-Values obtained of the differences between the second supernatant (glycosylated salivary alpha-amylase) activity after adding the methyl mannopyranoside at a sample dilution of 12.5 by Fisher’s test. The *p*-values in bold show significance. Figure S1: The original WBs in the two pools of the saliva samples shown in Figure 1.
