# Supplementary material for: Changes Occurring on the Activity of Salivary Alpha-Amylase Proteoforms in Two Naturalistic Situations Using a Spectrophotometric Assay
Source: Biology (Basel). 2021 Mar 16;10(3):227. doi: 10.3390/biology10030227 (PMC7999747; doi:10.3390/biology10030227)
Supplement: Supplementary file 1 [file biology-10-00227-s001.zip › Table S1, Supplementary data.pdf]

Changes occurring on the activity of salivary alpha-amylase proteoforms in two naturalistic situations using a spectrophotometric assay. María D. Contreras-Aguilar, Sandra V. Mateo, Fernando Tecles, Christophe Hirtz, Damián Escribano, Jose J. Cerón.

	**specimen 1**		**specimen 2**		**specimen 3**		***p* value[1]**
	**Activity measurement**	**NGsAA + GsAA**	**Activity measurement**	**NGsAA + GsAA**	**Activity measurement**	**NGsAA + GsAA**	
TsAA (IU/mL)	143.0		368.5		39.9		
NGsAA (IU/mL)	21.7		69.8		6.9		
GsAA (IU/mL)							
MMp treatment at 1:12.5	49.1	71.0	165.2	235.0	14.9	21.8	
MMp treatment at 1:25	66.7	88.5	217.0	286.8	19.2	26.1	0.286
MMp treatment at 1:50	76.5	98.3	267.9	337.6	26.0	32.9	0.060
MMp treatment at 1:100	91.4	113.2	285.8	355.6	26.8	33.7	**0.027**
MMp treatment at 1:200	89.4	111.3	300.0	369.8	27	33.9	**0.020**
