# Supplementary material for: Changes Occurring on the Activity of Salivary Alpha-Amylase Proteoforms in Two Naturalistic Situations Using a Spectrophotometric Assay
Source: Biology (Basel). 2021 Mar 16;10(3):227. doi: 10.3390/biology10030227 (PMC7999747; doi:10.3390/biology10030227)

Changes occurring on the activity of salivary alpha-amylase proteoforms in two naturalistic situations using a spectrophotometric assay. María D. Contreras-Aguilar, Sandra V. Mateo, Fernando Tecles, Christophe Hirtz, Damián Escribano, Jose J. Cerón.

Psychological stress model

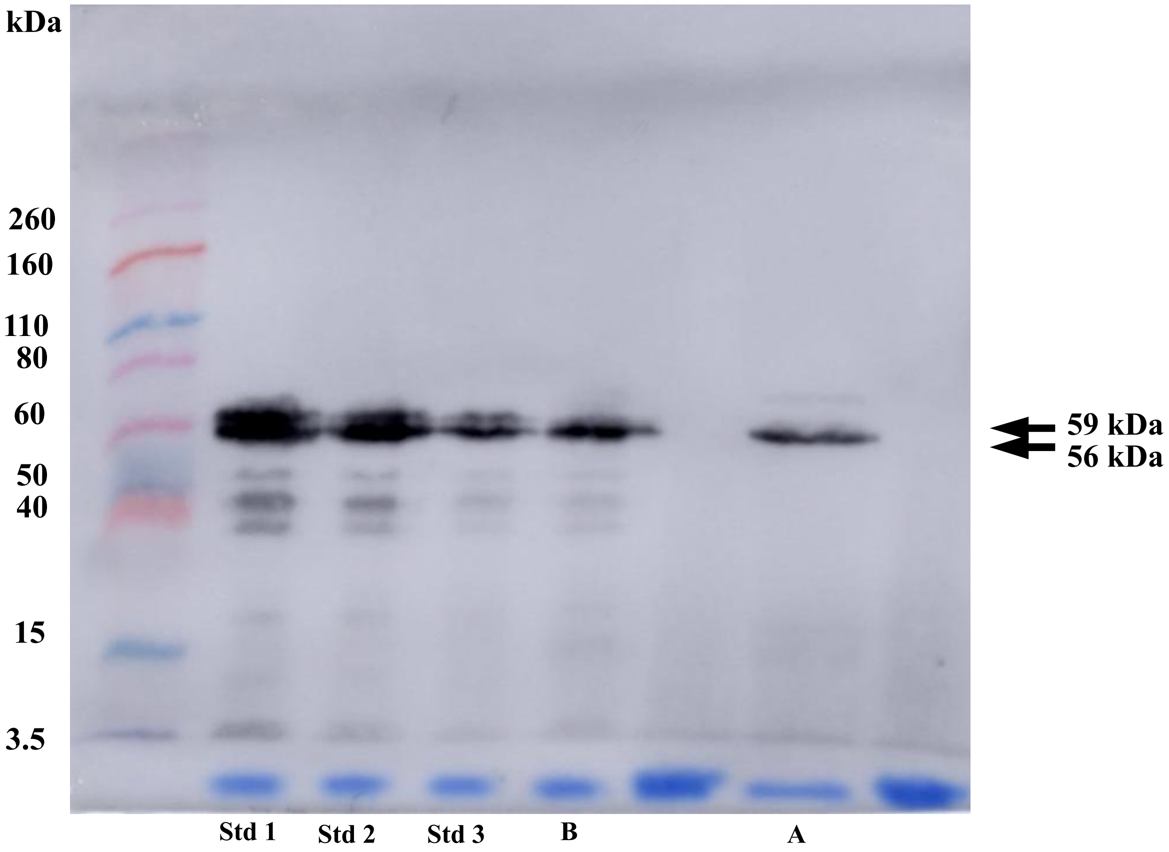

Physical stress model

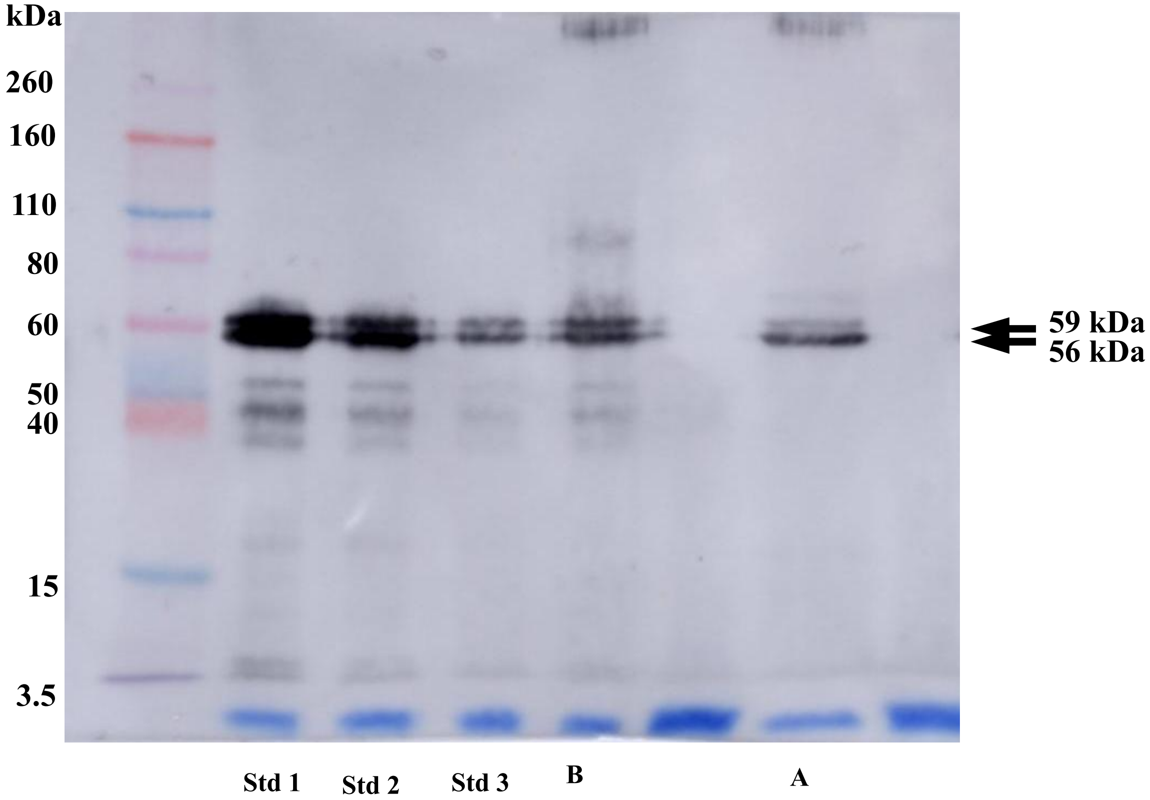

Supplement: Supplementary file 1 [file biology-10-00227-s001.zip › Figure S1, Supplementary data.pdf]
